# The Correlation of Serum Myeloid-Related Protein-8/14 and Eosinophil Cationic Protein in Patients with Coronary Artery Disease

**DOI:** 10.1155/2016/4980251

**Published:** 2016-02-28

**Authors:** Guo-lian Xia, Yun-kai Wang, Zhao-quan Huang

**Affiliations:** The Affiliated Hospital of Zhejiang Traditional Chinese Medicine University, Hangzhou 310006, China

## Abstract

*Objective*. To investigate the changes in serum Myeloid-Related Protein 8/14 (MRP8/14) and Eosinophil Cationic Protein (ECP) levels in patients with different types of coronary artery diseases (CAD) and assess the value of MRP8/14 and ECP detection in predicting CAD.* Methods*. 178 patients were divided into CAD group including unstable angina pectoris (UAP), acute myocardial infarction (AMI), and stable angina pectoris (SAP). Thirty-six individuals with normal coronary artery served as the control group. Serum MRP8/14 and ECP were measured by ELISA. The severity of coronary artery stenosis was assessed by the numbers of involved coronary artery branches and the sum of Gensini scores.* Results*. The MRP8/14 levels were significantly higher in AMI and UAP group than SAP and control group (*P* < 0.05). The levels of MRP8/14 in AMI group were also obviously higher than UAP group (*P* < 0.05). The ECP levels were obviously increased in AMI group, but there was no difference between SAP and UAP group (*P* > 0.05). The ECP was significantly increased in three impaired coronary arteries and obviously correlated with Gensini score (*P* < 0.01), whereas the MRP8/14 was obviously positively correlated with CRP (*P* < 0.01).* Conclusions*. Increased MRP8/14 levels suggest the instability of the atherosclerotic plaque. ECP reflects the severity of coronary arteries stenosis, predicting atherosclerosis burden. They may become the new biomarkers of CAD.

## 1. Introduction

Coronary artery disease (CAD) is rapidly increasing in prevalence across the world. The pathological basis of CAD is atherosclerosis (AS). Inflammation plays a key role in atherosclerotic plaque progression, vulnerability, and thrombogenicity [1]. So finding new biomarkers to reveal the atherosclerotic burden and the vulnerability of plaque are of great significance.

Myeloid-Related Protein 8/14 (MRP8/14), a heterodimer of two calcium binding proteins, has been implicated in the pathobiology of inflammatory disorders, such as inflammatory bowel disease, ankylosing spondylitis, and transplant rejection [2]. MRP8/14 broadly regulates vascular inflammation and contributes to the biological response to vascular injury by promoting leukocyte recruitment [3]. But the correlation of MRP8/14 and CAD is unclear.

Eosinophil Cationic Protein (ECP) is a member of the pancreatic-type extracellular ribonuclease (RNase) family, which has been extensively investigated as an efficacious biomarker of airway inflammation such as asthma [4]. ECP is a specific marker of eosinophils, reflecting the activation of eosinophil. It can stimulate effector cell to release the inflammatory mediators and exacerbates the inflammatory process [5]. However, the association of ECP with coronary atherosclerotic burden or with atherosclerotic plaque instability is controversial.

C-reactive protein (CRP), which is an inflammatory biomarker, has been widely studied in CAD [6]. In this study, we measured the serum levels of MRP8/14 and ECP compared to CRP in different types of coronary artery disease to investigate their association with the severity of CAD and traditional risk factors for coronary heart disease, exploring their value in the stratification of cardiovascular disease and prediction of the cardiovascular events.

## 2. Methods

### 2.1. Study Subjects

A total of 178 subjects were enrolled in the present study, which included 4 groups: the control group (*n* = 36), the stable angina pectoris group (SAP, *n* = 46), the acute coronary syndrome (ACS, *n* = 96) including the unstable angina pectoris group (UAP, *n* = 54), and the acute myocardial infarction (AMI, *n* = 42). All subjects were recruited from Zhejiang Traditional Chinese Medical Hospital in China from January 2014 to September 2014. All patients had undergone coronary angiography (CAG). The results of CAG were determined by experienced interventional cardiovascular interventional physicians. If coronary artery stenosis was more than 50%, the patients would be enrolled into the CAD group.

Exclusion criteria included any history of heart valve diseases, cardiomyopathy, cerebral vascular or peripheral vascular disease, myocarditis, pericarditis, left ventricular ejection fraction <35%, or overt congestive heart failure, autoimmune diseases, renal insufficiency, or cancer. And the diseases that cause the serum MRP8/14 elevation are not included in the scope of this study. 240 patients were recruited and 62 patients were excluded before and during this study. 20 patients were excluded as they developed into overt congestive heart failure and 13 patients were found with left ventricular ejection fraction <35% because of cardiomyopathy, myocarditis, and pericarditis. Eight patients had renal insufficiency and 6 patients had cancer by pathological diagnosis. Six patients had autoimmune diseases; 5 patients had cerebral vascular or peripheral vascular disease during this study.   10 patients died during this study; so they were excluded.

Carefully, all patients with cardiovascular risk factors were examined, including age, gender, diabetes (fasting blood glucose (FBS) > 126 mg/dL or treatment of diabetes), high blood lipids (total cholesterol > 200 mg/dL or treatment of high cholesterol HLP), smoking, and hypertension (received compression > 140 mmHg and/or diastolic blood pressure > 90 mmHg or treating hypertension).

Body mass index (BMI) and laboratory data including white blood cell (WBC), FBS, erythrocyte sedimentation rate (ESR), total cholesterol (TC), triglyceride (TG), high density lipoprotein cholesterol (HDL-C), low density lipoprotein cholesterol (LDL-C), Apolipoprotein (APOA), uric acid (UA), creatinine (Cr), blood urea nitrogen (BUN), and creatine kinase isoenzyme MB (CK-MB) fraction were also collected. The study protocol was approved by the ethics committee of our institution. All of the subjects enrolled in this study were Chinese and, after a complete explanation of the aims and details of the study, they had given informed consent to participate in this study.

### 2.2. Angiography

The severity of coronary artery stenosis was assessed by the numbers of involved coronary artery branches and the sum of the Gensini scores [7]. All patients underwent coronary angiography. Selective coronary angiography was performed with the technique of Judkins [8]. The angiographical severity of coronary stenosis was assessed in the worst view position, and the percentage of luminal narrowing was recorded according to the American Heart Association reporting system [9]. Patients with lesions of less than 50% luminal narrowing were defined as having no significant stenosis or 0-vessel disease (*n* = 36); coronary artery disease was defined as a stenosis of coronary artery of more than 50% in diameter in at least one branch and defined as a significant lesion (*n* = 142). The patients were referred to CAD as having single-vessel disease group (SVD, *n* = 41), double-vessel disease group (DVD, *n* = 56), or three or more diseased vessels (multivessel) group (MVD, *n* = 45). Four major coronary arteries and their main secondary branches were considered separately, that is, left main coronary artery (LM), left anterior descending artery (LAD), circumflex artery (LCX), and right coronary artery (RCA). The Gensini score was used to assess the severity of CAD: it graded narrowing of the lumen of the coronary artery and scored it as 1 for 1%–25% narrowing, 2 for 26%–50% narrowing, 4 for 51%–75% narrowing, 8 for 76%–90% narrowing, 16 for 91%–99% narrowing, and 32 for a completely occluded artery. This score was then multiplied by a factor according to the importance of the coronary artery. The multiplication factor for a LM lesion was 5; it was 2.5 for proximal LAD and LCX lesions, 1.5 for a mid-LAD lesion, and 1 for distal LAD, mid/distal LCX, and right coronary artery lesions. The multiplication factor for any other branch was 0.5.

### 2.3. Laboratory Tests

For the controls, venous blood samples were drawn on the day they came to have a medical examination with fasting. For CAD patients, venous blood samples were drawn from the antecubital vein after an overnight fast. For AMI patients, venous blood samples were drawn within 6 hours of admission. Serum samples were isolated by centrifugation for 15 minutes at 1500 g, aliquoted in 30 minutes, and stored at −80°C until analysis. We used chemiluminescent enzyme immunoassay (Access 2; Beckman Coulter, Chaska, Brea, CA, USA) for measurement of Troponin I (TnI) levels following the manufacturer's protocol. We analyzed the detection sensitivity, 20% coefficient of value (CV), and 10% CV value. We also used 0.04 ng/mL as the cutoff value for a positive diagnosis; that is, a positive elevation of TnI level was defined as TnI ≥ 0.04 ng/mL. C-reactive protein (CRP) was analyzed in a routine diagnostic analyzer using an ultrasensitive nephelometric method (DADE-Behring Latex BN-2), with a lower detection limit of 0.19 mg/L.

MRP8/14 concentration was assessed using a commercial sandwich enzyme-linked immunosorbent assay system (ELISA) development kit (R&D Systems, USA), an intra- and interassay coefficient of variance <9%, and a normal range of 3–320 pg/L, according to the manufacturer's instructions. The specific monoclonal antibody for the MRP8/14 heterodimer (mAb 27E10) was used as primary antibody and a polyclonal antibody coupled with horseradish peroxidase was used as secondary antibody. The antibody is specific for the MRP8/14 heterodimer or higher-order complexes and does not bind MRP8 or MRP14 monomers [10]. ECP was measured by a highly specific ELISA kit (R&D Systems, USA) and expressed as ng/L. The minimum detectable dose of ECP ranged from 5 to 200 ng/L and its interassay coefficient of variation was <9%. Absorbance was measured by a multimode detector (DTX880, Beckman Coulter, Fullerton, CA). A microplate reader capable of measuring absorbance at 450 nm was used.

### 2.4. Statistical Analysis

The distribution of continuous variables was determined by visual inspection of frequency histograms and with the use of the Shapiro-Wilk test. Continuous variables were expressed as mean ± standard deviation (SD) or median (interquartile range). Categorical variables were presented as ratio or constituent ratio. ANOVA was used to compare among multiple groups with continuous variables of normal distribution, in which LSD-*t* test was used for pairwise comparison; rank-sum test was used for nonnormal distribution data, in which Mann-Whitney* U* test was used for pairwise comparison. Categorical variables were compared by Chi-squared test.

Correlations between variables were performed by the Pearson test or Spearman's rank test, as appropriate. A multiple linear regression was performed to assess independent predictors of stenosis score and extent index. A two-tailed *P* value < 0.05 was the level of statistical significance. Statistical analyses were performed using SPSS statistical software (version 17.0, SPSS Inc., USA).

## 3. Results

### 3.1. Clinical Characteristics of the Study Population

The basic characteristics of the four groups are outlined in Table 1. Briefly, there were no statistical differences between age and gender, smoking, body mass index (BMI), hypertension, HbA1c, TG, TC, CHOL, CREA, BUN, and URIC (*P* > 0.05). However, differing with regard to cardiovascular risk factors which were more frequent in patients with CAD than control group, there were statistical differences between FBS, HDL-C, LDL-C, and APOA1 in these 4 groups (*P* < 0.05). And the levels of WBC and ESR were higher in patients with CAD than the controls (*P* < 0.05) and were the highest in AMI patients (*P* < 0.05).

### 3.2. Comparison of MRP8/14, ECP, and CRP Levels among Study Groups

**Figure 1:**
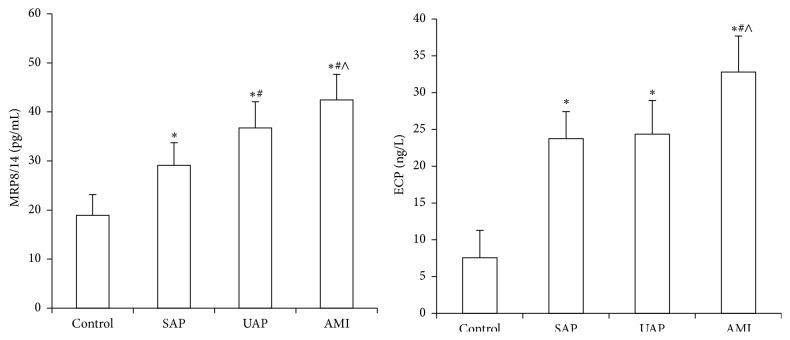
Comparison of the MRP8/14 and ECP in four groups. Compared with controls, ^*∗*^ *P* < 0.05; compared with SAP group, ^#^ *P* < 0.05; compared with UAP group, ^∧^ *P* < 0.05.

The serum MRP8/14 and ECP levels were significantly higher in CAD than the control group (*P* < 0.05), and the levels of MRP8/14 and ECP in AMI group elevated obviously higher than in UAP, SAP, and control group (*P* < 0.05). Compared with the SAP group, the MRP8/14 levels were significantly higher in UAP group [(29.12 ± 4.57) pg/mL versus (36.73 ± 5.34) pg/mL, *P* < 0.05]. But the ECP levels showed no statistical differences between SAP and UAP group [(23.73 ± 3.67) ng/L versus (24.36 ± 4.56) ng/L, *P* > 0.05]. The CRP levels in AMI and UAP group were significantly higher than SAP and control group [(23.38 ± 6.37) mg/L and (18.22 ± 5.44) mg/L versus (1.54 ± 0.55) mg/L and (0.19 ± 0.03) mg/L, *P* < 0.05], while there were no obvious differences between AMI and UAP group, as well as SAP and controls. The TnI levels elevated significantly higher in AMI patients, whereas there were no significant differences among UAP, SAP, and control group (Table 2, Figure 1).

### 3.3. Correlation between MRP8/14 and Other Factors

Simple analysis showed that MRP8/14 was positively correlated with WBC, CRP, CK-MB, and TnI (*P* < 0.05). These coefficients were clearly high for CRP (*r* = 0.535) and WBC (*r* = 0.364). No correlation was found between MRP8/14 and age, BMI, FBS, TC, HDL-C, LDL-C, TG, APOA1, BUN, CR, or UA (*P* > 0.05). MRP8/14 has no correlation with ECP (*P* > 0.05; Table 3).

Multiple linear regression analysis was performed to identify those variables independently related to MRP8/14. As shown in Table 3, WBC, cTnI, and CRP remained independently associated with MRP8/14 (Figure 3).

### 3.4. Correlation between ECP and Other Factors

Simple analysis showed that ECP was positively correlated with BMI, FBS, TC, TG, LDL-C, UA, and BUN (*r* > 0, *P* < 0.05), whereas a significantly negative correlation was observed with HDL-C (*r* = −0.292, *P* < 0.05) and APOA1 (*r* = −0.168, *P* < 0.05). These coefficients were clearly high for FBS (*r* = 0.421) and TC (*r* = 0.457). No correlation was found between ECP and age, Cr, CRP, WBC, CK-MB, and TnI (*P* > 0.05). Particularly, ECP and MRP8/14 have no correlation with each other (*P* > 0.05; Table 4). ECP remained independently associated with the Gensini score (Figure 4).

Multiple linear regression analysis was performed to identify those variables independently related to ECP. As shown in Table 4, FBS, TC, and HDL-C remained independently associated ECP.

### 3.5. The Levels of MRP8/14, ECP, and CRP in Different Groups according to the Number of ≥50% Stenotic Vessels

**Figure 2:**
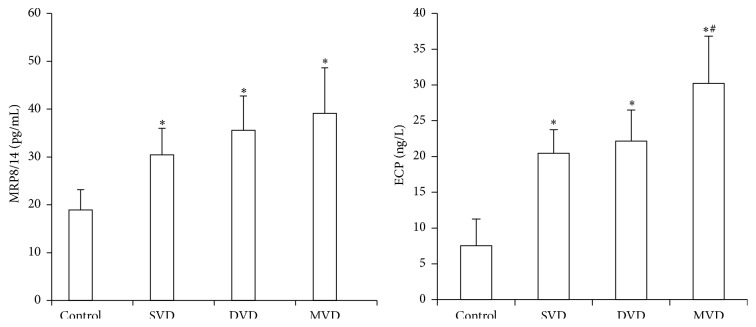
Comparison of the MRP8/14 and ECP in four groups. Compared with controls, ^*∗*^ *P* < 0.05; compared with SVD or MVD group, ^#^ *P* < 0.05.

The serum levels of ECP were significantly higher in three impaired coronary arteries than two vessels and one vessel impaired group (*P* < 0.05), but there were obviously positive correlations in levels of ECP with Gensini score (*P* < 0.05); however, there were no correlations in the levels of MRP8/14 and CRP with them (*P* > 0.05); that is, as the number of impaired vessels increased, the levels of MRP8/14 and CRP have not increased significantly (Table 5, Figure 2).

## 4. Discussion

Myeloid-Related Protein-8/14 (MRP8/14), also termed calprotectin, is mainly expressed in cells of myeloid origin [10], particularly in monocytes and neutrophils, but platelets [11], smooth muscle cells, and cardiac myocytes [12] also express this complex. MRP8 and MRP14 form stable complexes and represent the first cells invading inflammatory lesions, reflecting phagocyte activation and providing an enlarged mechanism of proinflammatory signals [13]. MRP8/14 combines TLR-4 on leukocytes, resulting in activation of nuclear factor-*κ*-B-regulated inflammatory responses and increasing expression of tumor necrosis factor-*α* [14]. MRP8/14 upregulates proinflammatory chemokines [15], such as IL-8, Gro-*α*, and MCP-1, and adhesion molecules such as VCAM-1 and ICAM-1. All of the above chemokines can promote further leukocyte recruitment and participate in the inflammatory pathobiology of atherosclerosis and plaque destabilization. Elevated serum levels of MRP8/14 also serve as an early and sensitive marker of myocardial necrosis in the setting of chest pain [16].

**Figure 3:**
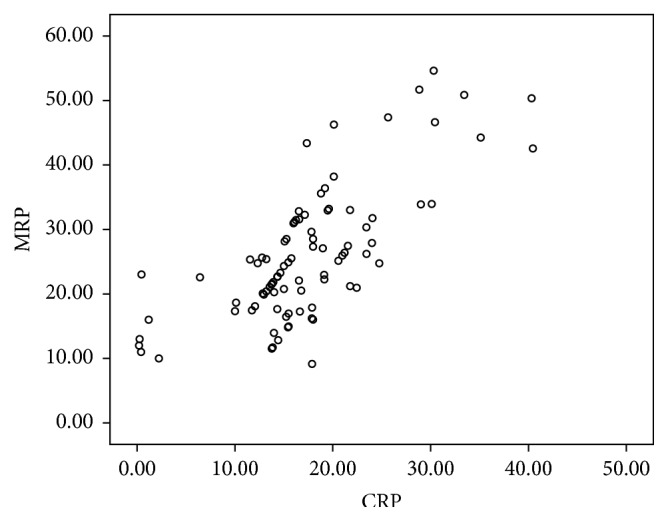
Correlation scatter diagram of MRP8/14 and hs-CRP (*r* = 0.731,  *P* < 0.05).

Troponin I (TnI) is considered as a specific biomarker of myocardial necrosis; it has facilitated diagnosis of AMI early, but it provides little information about unstable plaques. Some patients presenting with chest pain and negative Troponin experience cardiac events within the next 30 days [17]. MRP8/14 complex is highly expressed in human atherosclerotic lesions and CAD patients' circulating blood, which providing heightened inflammation and morphological features associated with plaque rupture [18]. In our study, the TnI levels were significantly elevated in AMI patients, whereas there was no significantly difference among UAP, SAP, and control group. On the contrary, the MRP8/14 levels in CAD patients were significantly elevated than control group, and the AMI patents had higher MRP8/14 levels than UAP patients, which was much higher than SAP group (Table 2, Figure 1), suggesting the MRP8/14 levels correlated with atherosclerotic plaque instability. The higher the MRP8/14 level, the more the instability in the plaque. MRP8/14 was positively and significantly correlated with WBC and CRP (Table 3, Figure 3), which represented inflammatory activation, demonstrating MRP8/14 was a key factor involved in the activation of inflammation. But the levels of CRP had no significant difference between AMI and UAP patients. So MRP8/14 was more sensitive than CRP in AMI patients. This study also showed MRP8/14 correlated with CK-MB and TnI (Table 3, Figure 3), which were the sensitive biomarkers of myocardial infarction. All the results consisted with the previous research. MRP8/14 triggered both a caspase-dependent and caspase-independent mechanism of cell death that accelerated myocardial cell death [19]. MRP8/14 was highly expressed at sites of coronary artery thrombosis and because serum levels of MRP8/14 elevated prior to myocardial necrosis markers, the molecule was a promising candidate biomarker for testing unstable plaques in the management of ACS [20]. The higher the MRP8/14 level was, the more easily the plaque ruptured and fell off, causing the occurrence of ACS. MRP8/14 may represent novel targets for anti-inflammatory strategies. However, the MRP8/14 levels had no correlation with the severity of coronary artery diseases and the extent of coronary lesions. MRP8/14 could not serve as an indicator to determine the severity of coronary artery disease. This study also confirmed that both the number of diseased vessels and coronary stenosis were not determinant factors of ACS, but the inflammation caused by vulnerable plaque and the severity of myocardial ischemia determined the occurrence of ACS.

**Figure 4:**
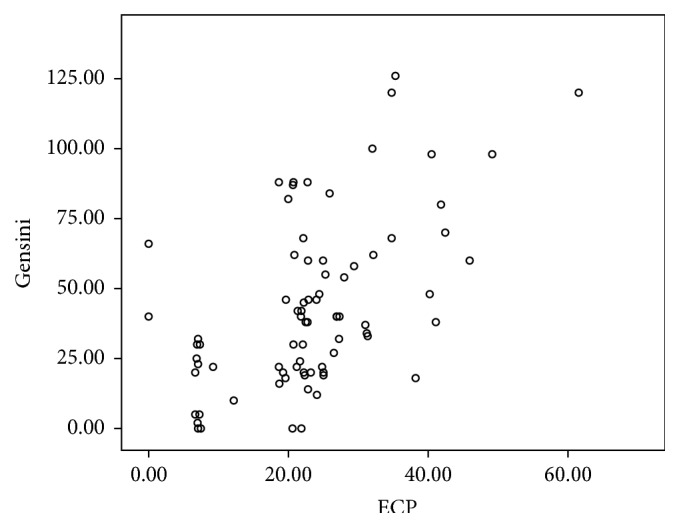
Correlation scatter diagram of ECP and Gensini (*r* = 0.546, *P* < 0.05).

Eosinophil Cationic Protein (ECP) is a zinc-containing, highly cationic protein and stored in the peroxidase-positive and peroxidase-negative eosinophil granules. ECP increases TNF-*α* production and triggers apoptosis by caspase-8 activation through mitochondria-independent pathway in BEAS-2B cells [21]. ECP induced coagulation cascade reaction and resulted in the enhancement of fibrinolytic activity. Coronary heart disease patients confirmed by coronary angiography had higher levels of eotaxin compared with the control group, which was a specific type of eosinophils [22]. ECP levels were expressed higher in the CAD group. The levels of ECP in three impaired vessels' group were significantly elevated than one and two impaired vessels' group, and there were no significant differences between one and two impaired vessels' group (Tables 2 and 5, Figure 2). And there was a significantly positive correlation between ECP concentrations and the Gensini score (Table 4, Figure 4). ECP was positively correlated with BMI, FBS, TC, TG, TC, LDL-C, UA, and BUN (*r* > 0, *P* < 0.05), whereas a significantly negative correlation was observed with HDL-C and APOA1 (Table 4). No correlation was found between ECP and age, Cr, CRP, WBC, CK-MB, and TnI (*P* > 0.05; Table 4). Particularly, ECP and MRP8/14 had no correlation with each other. Integrated, all the results demonstrated that ECP could reflect the growth of atherosclerotic plaque but could not reflect the plaque instability, which consisted with ECP modulated fibroblast activity, increasing collagen release and effectively stabling on plaque growth [23]. Emanuele et al. [24] demonstrated a positive correlation between the number of diseased coronary arteries and the serum levels of eotaxin, thus indicating eosinophils may be involved in the formation of coronary atherosclerotic burden. C-reactive protein (CRP), a nonspecific marker of inflammation, which had no correlation with ECP in this study, was most widely studied and associated with increasing risk of future cardiovascular events. CRP levels associate with atherosclerotic plaque instability, but they cannot reflect coronary atherosclerotic burden [25]. ECP could not prompt the development of ACS. However, when combined with major cardiovascular factors, ECP could improve the stratification performance of risk factors for diagnosis of coronary atherosclerosis by coronary angiography in patients with chest pain.

MRP8/14 and ECP, the two serum biochemical parameters, have the similarities to assess coronary heart disease; that is, the expression of MRP8/14 and ECP in CAD patients is significantly increased; they play important role in the stratification of risk factors in coronary heart disease. The difference is that the MRP8/14 levels are significantly higher in UAP than SAP patients, but there is no significant difference in the ECP levels between the two groups. It shows the rise in serum ECP levels has nothing to do with plaque instability and cannot reflect urgency in CAD patients but prompt the growth of plaque. ECP reflects the severity of coronary artery stenosis. MRP8/14 has no obvious correlation with the degree of coronary stenosis; its elevating levels are able to predict plaque instability, indicating acute myocardial infarction or unstable angina pectoris. MRP8/14 has an early diagnosis value of coronary heart disease. Therefore, MRP8/14 and ECP are expected to become new biomarkers with coronary heart disease, and joint detection can improve the diagnosis of coronary heart disease, the development of disease, and prognosis of clinical applications.

## 5. Conclusions

The increased serum levels of MRP8/14 and CRP in CAD aggravate the development of atherosclerosis and mediate the rupture of instable plaque. ECP was associated with plaque growth and reflected the severity of coronary arteries stenosis. They may become the new biomarkers of CAD.

## Figures and Tables

**Table 1:** Characteristics of SAP, UAP, and AMI patients and controls.

Basic information	Control (*n* = 36)	SAP (*n* = 46)	UAP (*n* = 54)	AMI (*n* = 42)
Ages (years)	62.00 ± 10.93	73.13 ± 8.25	73.24 ± 9.06	66.85 ± 11.52
Gender (M/F)	26/10	35/11	40/14	32/10
Smoking (Y/N)	12/24	31/15	34/20	27/15
Diabetes (Y/N)	5/31	10/36	21/33	12/30
Hypertension (Y/N)	11/25	24/22	40/14	30/12
Dyslipidemia (Y/N)	16/20	27/19	39/15	28/14
BMI (kg/m^2^)	23.29 ± 2.57	25.18 ± 3.12	25.82 ± 3.64	24.65 ± 1.87
WBC (×10^9^/L)	5.18 ± 0.81	5.83 ± 1.63	6.04 ± 2.09^*∗*^	8.45 ± 4.85^*∗*#△^
ESR (mm/h)	8.20 ± 3.33	14.46 ± 5.45	20.34 ± 6.52	29.00 ± 7.47^*∗*^
FBS (mmol/L)	5.29 ± 0.99	5.44 ± 1.00	5.74 ± 1.23	6.37 ± 1.77^*∗*^
HbA1C (%)	5.80 ± 0.26	6.60 ± 1.03	9.23 ± 1.66	7.63 ± 1.71
TG (mmol/L)	1.57 ± 0.28	1.54 ± 0.17	1.64 ± 0.20	1.99 ± 0.65
TC (mmol/L)	4.60 ± 1.16	4.12 ± 0.81	3.71 ± 1.31	3.99 ± 0.96
HDL-C (mmol/L)	1.74 ± 0.10	1.49 ± 0.36	1.46 ± 0.39	1.30 ± 0.31^*∗*^
LDL-C (mmol/L)	2.52 ± 10.97	2.07 ± 0.67	1.64 ± 0.80^*∗*^	1.90 ± 0.73
APOA1 (g/L)	1.47 ± 0.16	1.34 ± 0.22	1.27 ± 0.20^*∗*^	1.16 ± 0.20^*∗*^
UA (*μ*mol/L)	298.60 ± 66.12	338.92 ± 108.33	320.53 ± 98.60	359.70 ± 96.02
Cr (*μ*mol/L)	57.56 ± 9.27	75.79 ± 29.67	78.18 ± 30.80	77.05 ± 32.91
BUN (mmol/L)	6.22 ± 1.34	6.20 ± 1.31	6.51 ± 180	7.62 ± 5.21

AMI, acute myocardial infarction; UAP, unstable angina pectoris; SAP, stable angina pectoris; BMI, body mass index; WBC, white blood cell; FBS, fasting blood sugar; TC, total cholesterol; TG, triglyceride; HDL-C, high density lipoprotein cholesterol; LDL-C, low density lipoprotein cholesterol; APOA: Apolipoprotein; UA, uric acid; Cr, creatinine; BUN, blood urea nitrogen; CK-MB, creatine kinase isoenzyme MB fraction; CRP, C-reactive protein; TnI, Troponin I; MRP8/14, Myeloid-Related Protein 8/14; ECP, eosinophil cationic protein.

*P* value was the comparing results of the 4 groups.

^*∗*^ *P* < 0.05 compared with the control group; ^#^ *P* < 0.05 compared with the SAP group; ^△^ *P* < 0.05 compared with the UAP group.

**Table 2:** MRP8/14, ECP, and CRP of SAP, UAP, and AMI patients and controls.

Basic information	Control (*n* = 36)	SAP (*n* = 46)	UAP (*n* = 54)	AMI (*n* = 42)
CK-MB (IU/L)	9.40 ± 2.34	10.88 ± 3.56	10.75 ± 3.17	37.30 ± 5.69^*∗*#∧^
TnI (*μ*g/L)	0.08 ± 0.03	0.37 ± 0.08	0.54 ± 0.17	27.6 ± 15.28^*∗*#∧^
CRP (mg/L)	0.19 ± 0.03	1.54 ± 0.55	18.22 ± 5.44^*∗*#^	23.38 ± 6.37^*∗*#^
MRP8/14 (pg/mL)	18.92 ± 4.23	29.12 ± 4.57^*∗*^	36.73 ± 5.34^*∗*#^	42.45 ± 5.21^*∗*#∧^
ECP (ng/L)	7.53 ± 3.73	23.73 ± 3.67^*∗*^	24.36 ± 4.56^*∗*^	32.78 ± 4.91^*∗*#∧^

Abbreviations as shown in Table 1.

*P* value was the comparing results of the 4 groups.

^*∗*^ *P* < 0.05 compared with the control group; ^#^ *P* < 0.05 compared with the SAP group; ^∧^ *P* < 0.05 compared with the UAP group.

**Table 3:** Simple and multiple regression analyses between MRP8/14 and other factors.

Basic information	Simple regression		Multiple regression	
	*r*	*P*	*β*	*P*
Ages (years)	0.006	0.933		
BMI (kg/m2)	0.052	0.488		
WBC (×10^9^/L)	0.364	0.000	0.187	0.030
FBS (mmol/L)	0.030	0.685		
HbA1c (%)	0.045	0.529		
TG (mmol/L)	0.124	0.094		
TC (mmol/L)	0.024	0.741		
HDL-C (mmol/L)	−0.102	0.171		
LDL-C (mmol/L)	0.048	0.516		
APOA1 (g/L)	−0.015	0.838		
BUN (*μ*mol/L)	−0.062	0.405		
Cr (*μ*mol/L)	0.005	0.946		
UA (umol/L)	−0.012	0.873		
CK-MB (IU/L)	0.146	0.049		
CRP (mg/L)	0.731	0.000	0.263	0.006
TnI (*μ*g/L)	0.291	0.000	0.146	0.021
ECP (ng/L )	0.079	0.243		

Abbreviations as shown in Table 1.

The *r* value was the coefficient correlation; *P* value was the significance test of coefficient correlation.

**Table 4:** Simple and multiple regression analyses between ECP and other factors.

Basic information	Simple regression		Multiple regression	
	*r*	*P*	*β*	*P*
Ages (years)	0.084	0.261		
BMI (kg/m^2^)	0.180	0.000		
WBC (×10^9^/L)	0.072	0.342		
FBS (mmol/L)	0.421	0.000	0.235	0.003
HbA1c (%)	0.145	0.056		
TG (mmol/L)	0.322	0.000		
TC (mmol/L)	0.457	0.000	0.242	0.001
HDL-C (mmol/L)	−0.292	0.000	−0.156	0.034
LDL-C (mmol/L)	0.172	0.005		
APOA1 (g/L)	−0.168	0.007		
BUN (umol/L)	0.152	0.043		
Cr (*μ*mol/L)	0.155	0.042		
UA (*μ*mol/L)	0.091	0.238		
CK-MB (IU/L)	0.116	0.159		
CRP (mg/L)	0.139	0.080		
TnI (*μ*g/L)	0.140	0.077		
MRP8/14 (pg/mL)	0.086	0.243		
Gensini score	0,546	0.000		

Abbreviations as shown in Table 1.

The *r* value was the coefficient correlation; *P* value was the significance test of coefficient correlation.

**Table 5:** The levels of MRP8/14, ECP, and CRP in different groups according to the number of ≥50% stenotic vessels.

Impaired vessel count	*N*	MRP8/14 (pg/mL)	ECP (ng/L)	CRP (mg/L)
Control	36	18.92 ± 4.23	7.53 ± 3.73	0.19 ± 0.03
SVD	41	30.45 ± 5.54^*∗*^	20.45 ± 3.28^*∗*^	15.76 ± 0.14
DVD	56	35.57 ± 7.17^*∗*^	22.17 ± 4.33^*∗*^	18.97 ± 5.15
MVD	45	39.11 ± 9.52^*∗*^	30.21 ± 6.59^*∗*#^	25.11 ± 7.21^*∗*#^

Abbreviations as shown in Table 1.

^*∗*^ *P* < 0.05 compared with the control group; ^#^ *P* < 0.05 compared with the SVD and DVD group.
